# Secondary traumatic stress and posttraumatic growth in newly graduated nurses: the mediating role of compassion satisfaction

**DOI:** 10.1186/s12912-023-01456-w

**Published:** 2023-08-31

**Authors:** Li Zeng, Xiangeng Zhang, Guiling Liu, Dong Liu, Lan Li, Man Jin, Xin Li, Jialin Wang

**Affiliations:** 1 Sichuan Nursing Vocational College, Chengdu, Sichuan China; 2 College of Nursing, Chengdu University of Traditional Chinese Medicine, Chengdu, Sichuan China; 3 College of Modern Nursing, Dazhou Vocational and Technicial College, Dazhou, Sichuan China; 4 School of Pharmacy, Chengdu Medical College, Chengdu, Sichuan China; 5 The Third People’s Hospital of Chengdu, Chengdu, Sichuan China; 6 Army Medical Center of PLA, Chongqing, China

**Keywords:** Newly graduated nurses, Secondary traumatic stress, Compassion satisfaction, Posttraumatic growth

## Abstract

**Background:**

Nurses’ secondary traumatic stress, compassion satisfaction and posttraumatic growth are closely related, but for newly graduated nurses, there are few reports to evaluate the specific path between these three. The aim of this study was to investigate examine the mediating role of compassion satisfaction in secondary traumatic stress and posttraumatic growth among newly graduated nurses.

**Methods:**

From March 2021 to May 2021, a total of 330 newly graduated nurses from five tertiary hospitals in China were enrolled, and asked to complete questionnaires regarding secondary traumatic stress, compassion satisfaction and posttraumatic growth. Descriptive statistics, independent-samples T-test, one-way analysis of variance (ANOVA), Pearson correlation analysis and structural equation model were used in this study. A STROBE checklist was used to report findings.

**Results:**

The scores of secondary traumatic stress, compassion satisfaction and posttraumatic growth of newly graduated nurses were 27.11 ± 4.94 (score range: 10–50), 31.89 ± 6.22 (score range: 10–50) and 56.47 ± 20.41 (score range: 0-100), respectively. Additionally, structural equation modeling showed that compassion satisfaction mediated the relationship between secondary traumatic stress and posttraumatic growth with the partial mediating effect of 0.089.

**Conclusions:**

Newly graduated nurses may experience moderate secondary traumatic stress, but their posttraumatic growth is at a low level, and compassion satisfaction significantly affects the relationship between the two. Nursing managers should strengthen psychological evaluation, and promote their posttraumatic growth by improving their level of compassion satisfaction.

## Introduction

The acceleration of the global population aging process has put forward higher service requirements for the medical and health care industry, while nurses, as important medical and health care personnel, are facing a shortage of personnel, which is undoubtedly a great threat to the quality of medical and health care services in various countries [1]. Some studies pointed out that recruiting newly graduated nurses was one of the effective ways to supplement nursing staff and expand the nursing team, which also helps to alleviate the continuous crisis of health care services [2, 3]. However, newly graduated nurses may experience a gap between theory and practice when they first enter the clinical environment [4]. An effective transition plan is an important support system for newly graduated nurses to pass the transition period, which is important and complex for nursing managers, such as standardized training for nurses in China [5]. Standardized training for nurses refers to nurses receiving standardized and professional nursing theory and practical training after completing basic education in medical colleges, and standardized training for nurses is an important way to cultivate a nursing talent team, and an important measure to enhance nurses’ comprehensive abilities, improve clinical nursing quality [6]. Nevertheless, due to the high risk and pressure of work in the nursing industry, newly graduated nurses may often encounter considerable challenges when transitioning to clinical practice at the initial stage of employment because of their lack of clinical work experience, such as secondary traumatic stress [7].

Secondary traumatic stress refers to a natural stressful behavior and psychological emotional result produced by the group of helpers who are indirectly exposed to the injured and their painful experiences in the process of helping or wanting to help the injured and the suffering [8]. Mangoulia et al. believed that for nurses, secondary traumatic stress was an occupational hazard, which is not only detrimental to the physical and mental health of nurses, but also reduces the ability of nurses to provide nursing services and make professional judgments, seriously affecting the personal life and work of nurses [9]. Wang et al. also pointed out that nurses with high levels of secondary traumatic stress might have emotional exhaustion and turnover intention, which directly affects the quality of clinical nursing service and the stability of nursing team [10]. Moreover, some studies showed that newly graduated nurses reported significantly higher levels of secondary traumatic stress than experienced senior nurses [3, 11]. Therefore, it is necessary to pay attention to the psychological stress response of newly graduated nurses after indirect trauma exposure.

Despite experiencing indirect trauma exposure will cause nurses to have negative psychological stress response, such as secondary traumatic stress, positive psychological changes may also occur, such as posttraumatic growth. Posttraumatic growth refers to the positive psychological changes experienced by individuals in the fight against highly challenging life events and traumatic events, including changes in self-awakening, interpersonal relations and life philosophy [12]. Relevant studies confirmed that the occurrence of posttraumatic growth could enhance nurses’ ability to adapt to the environment, which is conducive to alleviating nurses’ work pressure, and improving their job satisfaction and subjective well-being [13, 14]. As for the influencing factors of nurses’ posttraumatic growth, some studies suggested that secondary traumatic stress might become a catalyst for posttraumatic growth, that is, the higher the level of secondary traumatic stress, the stronger the nurses’ self-awakening and determination to seek change, and the higher the level of posttraumatic growth [8, 15]. Another study found that compassion satisfaction was also an important factor affecting nurses’ posttraumatic growth [16].

Compassion satisfaction means that nurses can obtain a sense of pleasure and achievement by providing nursing services to patients [10]. Peters believed that compassion satisfaction could provide energy, insight and firm determination for nursing services, so as to promote nurses to show positive work attitude, high-level professional value and high-quality nursing services [17]. Yu, Jiang & Shen proposed that a high level of compassion satisfaction could not only improve the quality of nursing services, but also prolong the professional life of nurses and reduce the risk of nurses leaving [18]. Moreover, some studies pointed out that compassion satisfaction could be used as a regulating or buffering factor of compassion fatigue, and the higher the level of compassion satisfaction of nurses, the stronger their ability to cope with psychological stress, and the greater their relief effect on secondary traumatic stress and burnout, which is conducive to promoting the posttraumatic growth of nurses [10, 19].

Although previous studies have shown that there is a correlation between nurses’ secondary traumatic stress, compassion satisfaction and posttraumatic growth, few studies have investigated the mediating role of compassion satisfaction on the relationship between secondary traumatic stress and posttraumatic growth, especially for newly graduated nurses. Therefore, in order to provide theoretical basis for nursing managers to formulate targeted measures to improve the mental health level of newly graduated nurses, this study aims to clarify the relationship between secondary traumatic stress and posttraumatic growth of newly graduated nurses, and explore the mediating role of compassion satisfaction between the two.

This study mainly proposes the following hypothesis:

### Hypothesis 1

Newly graduated nurses’ secondary traumatic stress is closely related to their posttraumatic growth.

### Hypothesis 2

Newly graduated nurses’ compassion satisfaction is closely related to their posttraumatic growth.

### Hypothesis 3

The relationship between newly graduated nurses’ secondary traumatic stress and posttraumatic growth is mediated by compassion satisfaction.

## Methods

### Aim

This study aimed to explore the relationship between secondary traumatic stress and posttraumatic growth, as well as the mediating role of compassion satisfaction in this relationship among newly graduated nurses working in Chengdu, China.

### Design

This was a cross-sectional study, and to ensure the research quality, the STROBE checklist was used to report findings.

### Participants

From March 2021 to May 2021, participants were selected from five tertiary grade A hospitals in Chengdu, Sichuan Province, China by convenient sampling. The inclusion criteria of participants were: (a) registered nurses, (b) with < 1 year of clinical nursing experience, and (c) volunteered to participation in this study. Exclusion criteria: (a) non formal staff of the investigated hospital, such as refresher nurses; (b) Those who are not on duty at the time of investigation. These participants work in different departments, including internal medicine, surgery, pediatrics, oncology, psychiatry, ICU, etc.

### Data collection

First of all, we contacted the heads of the relevant management departments of each hospital and obtained the investigation permission. Then we sent the electronic questionnaire through the network platform to conduct a pre survey, and conducted cultural debugging on the total table through the feedback of the pre survey. After the pre survey, the final version of the electronic questionnaire was distributed again through the network platform, including a unified guide, which explained the purpose of the survey, the definition of survey related variables and precautions for filling in, and pointed out that the survey was anonymous and voluntary. According to the literature, the sample size should be 5–10 times the number of items [20, 21]. In addition, considering the possible quality problems in the questionnaire, the sample size should be expanded by 20%. Therefore, our study requires at least 282 participants. Finally, a total of 350 questionnaires were distributed and 330 valid questionnaires were recovered, with a response rate of 94.29%.

### Measures

#### Secondary traumatic stress

To assess secondary traumatic stress of nurses, the Chinese version Professional Quality of Life Scale (ProQOL-CN), which is a translated and revised by Zheng et al. of the original ProQOL, developed by Stamm, was used [22, 23]. The ProQOL-CN has three sub scales, namely, compassion satisfaction (CS), burnout (BO) and secondary traumatic stress (STS), and each sub scale has 10 items, a total of 30 items. Among the 10 items used to measure secondary traumatic stress, participants were asked to answer with a 5-point Likert scale, ranging from 1 (“never”) to 5 (“always”), and the higher the score, the higher the nurse’s secondary traumatic stress level. It was generally believed that a score ≤ 22 is a low level, 23–41 is a moderate level, and ≥ 42 is a high level [22, 23]. Regarding the reliability of the secondary traumatic stress sub scale, the Cronbach’s α of the ProQOL was 0.81, and in this study, the Cronbach’s α was 0.80.

#### Compassion satisfaction

To assess compassion satisfaction of nurses, the ProQOL-CN was used. Among the compassion satisfaction sub scale, participants were asked to answer with a 5-point Likert scale, ranging from 1 (“never”) to 5 (“always”), and the higher the score, the higher the nurse’s compassion satisfaction level. Similarly, the score ≤ 22 is a low level, 23–41 is a moderate level, and ≥ 42 is a high level [22, 23]. Regarding the reliability of the compassion satisfaction sub scale, the Cronbach’s α of the ProQOL was 0.88, and in this study, the Cronbach’s α was 0.91.

#### Posttraumatic growth

To assess posttraumatic growth of nurses, the Simplified Chinese version of the Posttraumatic Growth Inventory (C-PTGI), which is a translated and revised by Wang et al. of the original PTGI, developed by Tedeschi & Calhoun, was used [24, 25]. C-PTGI includes five subscales, namely, relating to others, new possibilities, personal strength, spiritual change, and insights on life, with a total of 20 items (the original 18th item “I have a stronger religious faith” was deleted). Participants were asked to answer with a 6-point Likert scale, ranging from 0 (no change) to 5 (great change), and the higher the score, the higher the nurse’s posttraumatic growth level. Among them, the score < 60 is a low growth level, 60–65 is a moderate growth level, and > 65 is a high growth level [25]. Regarding the reliability, the Cronbach’s α of the PTGI was 0.90 and for the subscales was 0.67 to 0.85, and in this study, the Cronbach’s α was 0.97 and for the subscales was 0.89 to 0.93.

### Data analysis

SPSS 26.0 and AMOS 26.0 were used to analyze the data. Descriptive statistics were used to present the social-demographic information of participants. Since our data approximate the normal distribution, independent sample *t*-test or one-way analysis of variance (ANOVA) were used to test the scores of secondary traumatic stress, compassion satisfaction and posttraumatic growth of newly graduated nurses with different social-demographic information. Pearson correlation analysis was used to evaluate the relationship between variables. Structural equation model was used to explore the relationship between secondary traumatic stress, compassion satisfaction and posttraumatic growth of newly graduated nurses, and to test the mediating role of compassion satisfaction. In the study, judge whether the model fitting is reasonable by *χ2*/df, Tacker-Lewis index (TLI), comparative fit index (CFI), incremental fit index (IFI), relative fit index (RFI), normal fit index (NFI), and root mean square error of approximation (RMSEA). The *χ*^*2*^/df < 5, TLI, CFI, IFI, RFI and NFI > 0.90, RMSEA ≤ 0.08 are considered to be reasonable model fitting. In addition, *p* < 0.05 was considered statistically significant (two-tailed test).

### Ethical consideration

The principles of anonymity and informed consent were strictly followed throughout the study, and this study has been approved by the Ethics Committee of Chengdu University of Traditional Chinese Medicine (Number: 2020-KL084).

## Results

### Participant characteristics

Of the 330 newly graduated nurses, 7.88% were males and 92.12% were females, with a mean age of 22.73 ± 1.89. Only 7.58% of newly graduated nurses were married. The majority of newly graduated nurses had bachelor degree or above (62.73%), were on day shift (64.24%), worked hours per day > 8 h (65.76%), slept hours per day > 7 h (65.76%). More than half (59.70%) of newly graduated nurses had workplace violence experience (Table 1).

**Table 1:** Demographic characteristics and scores of secondary traumatic stress, compassion satisfaction and posttraumatic growth (N = 330)

Variables	N (%)	Secondary traumatic stress Mean (SD)	Compassion satisfaction Mean (SD)	Posttraumatic growth Mean (SD)
Gender				
Male	26 (7.88)	27.54 (6.94)	32.35 (7.73)	56.12 (19.42)
Female	304 (92.12)	27.07 (4.74)	31.85 (6.09)	56.50 (20.52)
*t* (*p*)		0.216 (0.642)	0.153 (0.696)	0.009 (0.926)
Education level				
Associate degree or less	123 (37.27)	27.32 (5.14)	32.15 (6.77)	54.54 (22.35)
Bachelor degree or above	207 (62.73)	26.98 (4.82)	31.73 (5.88)	57.62 (19.12)
*t* (*p*)		0.358 (0.550)	0.359 (0.549)	1.769 (0.184)
Marital status				
Married	25 (7.58)	28.56 (4.95)	33.08 (6.58)	56.72 (24.05)
Member of an unmarried couple	146 (44.24)	26.73 (4.73)	31.83 (5.88)	56.95 (20.37)
Single/Divorced or separated	159 (48.18)	27.23 (5.10)	31.75 (6.49)	56.00 (19.95)
*F* (*p*)		1.570 (0.210)	0.500 (0.607)	0.083 (0.920)
Departments				
Internal medicine	49 (14.85)	27.94 (5.19)	32.00 (7.07)	61.18 (19.08)
Surgery	62 (18.79)	26.58 (4.29)	29.79 (5.03)	54.55 (20.91)
Pediatrics	24 (7.27)	25.63 (5.87)	31.17 (7.26)	55.96 (23.95)
Oncology	15 (4.54)	27.07 (3.58)	32.80 (4.00)	56.40 (19.45)
Psychiatry	85 (25.76)	27.54 (5.02)	32.29 (6.41)	52.40 (19.97)
ICU	39 (11.82)	27.62 (4.79)	33.21 (6.06)	61.51 (20.81)
Others	56 (16.97)	26.59 (5.21)	32.64 (6.17)	57.39 (19.39)
*t* (*p*)		0.991 (0.431)	1.800 (0.099)	1.524 (0.170)
Scheduling				
Day shift	212 (64.24)	26.71 (4.80)	32.26 (6.12)	57.37 (20.33)
Night shift	118 (35.76)	27.81 (5.12)	31.21 (6.38)	54.86 (20.54)
*t* (*p*)		3.806 (0.052)	2.176 (0.141)	1.153 (0.284)
Work hours per day				
≤ 8 h	113 (34.24)	25.89 (4.91)	32.59 (6.79)	56.09 (21.80)
> 8 h	217 (65.76)	27.74 (4.84)	31.52 (5.89)	56.67 (19.69)
*t* (*p*)		**10.670 (0.001)**	2.215 (0.138)	0.061 (0.805)
Sleep hours per day				
≤ 7 h	113 (34.24)	27.93 (5.66)	30.78 (6.77)	52.29 (20.22)
> 7 h	217 (65.76)	26.68 (4.47)	32.47 (5.85)	58.65 (20.21)
*t* (*p*)		**4.835 (0.029)**	**5.537 (0.019)**	**7.351 (0.007)**
Workplace violence experience				
Yes	197 (59.70)	27.60 (4.86)	31.54 (6.31)	54.47 (20.48)
No	133 (40.30)	26.38 (4.97)	32.40 (6.08)	59.44 (20.01)
*t* (*p*)		**4.933 (0.027)**	1.503 (0.221)	**4.776 (0.030)**

### The level of secondary traumatic stress, compassion satisfaction and posttraumatic growth

The scores of secondary traumatic stress, compassion satisfaction and posttraumatic growth of newly graduated nurses were 27.11 ± 4.94, 31.89 ± 6.22 and 56.47 ± 20.41, respectively (Table 2). Moreover, newly graduated nurses who worked hours per day > 8 h, slept hours per day ≤ 7 h and had workplace violence experience reported higher secondary traumatic stress scores. Newly graduated nurses who slept hours per day > 7 h reported higher compassion satisfaction, while newly graduated nurses who slept hours per day ≤ 7 h and had workplace violence experience reported lower posttraumatic growth scores (*p* < 0.05) (Table 1).

### Correlations of secondary traumatic stress, compassion satisfaction and posttraumatic growth

Pearson correlation analysis suggested that secondary traumatic stress and compassion satisfaction of newly graduated nurses were significantly positively associated with posttraumatic growth and its five subscales (*p* < 0.01) (Table 2).

**Table 2:** Means, standard deviations, and correlations of secondary traumatic stress, compassion satisfaction and posttraumatic growth (N = 330)

Variable	Mean (SD)	1	2	3	4	5	6	7	8
1. Secondary traumatic stress	27.11 (4.94)	1							
2. Compassion satisfaction	31.89 (6.22)	0.160^**^	1						
3. Posttraumatic growth	56.47 (20.41)	0.199^**^	0.565^**^	1					
4. Relating to others	19.03 (7.29)	0.167^**^	0.569^**^	0.957^**^	1				
5. New possibilities	13.04 (5.28)	0.184^**^	0.522^**^	0.959^**^	0.889^**^	1			
6. Personal strength	11.06 (4.10)	0.154^**^	0.557^**^	0.953^**^	0.883^**^	0.911^**^	1		
7. Spiritual change	4.48 (2.15)	0.238^**^	0.339^**^	0.735^**^	0.642^**^	0.659^**^	0.641^**^	1	
8. Insights on life	8.86 (3.28)	0.221^**^	0.491^**^	0.880^**^	0.777^**^	0.809^**^	0.831^**^	0.625^**^	1

^*^p < 0.05, ^**^p < 0.01

### The mediating role of compassion satisfaction on the relationships between secondary traumatic stress and posttraumatic growth

**Fig. 1:**
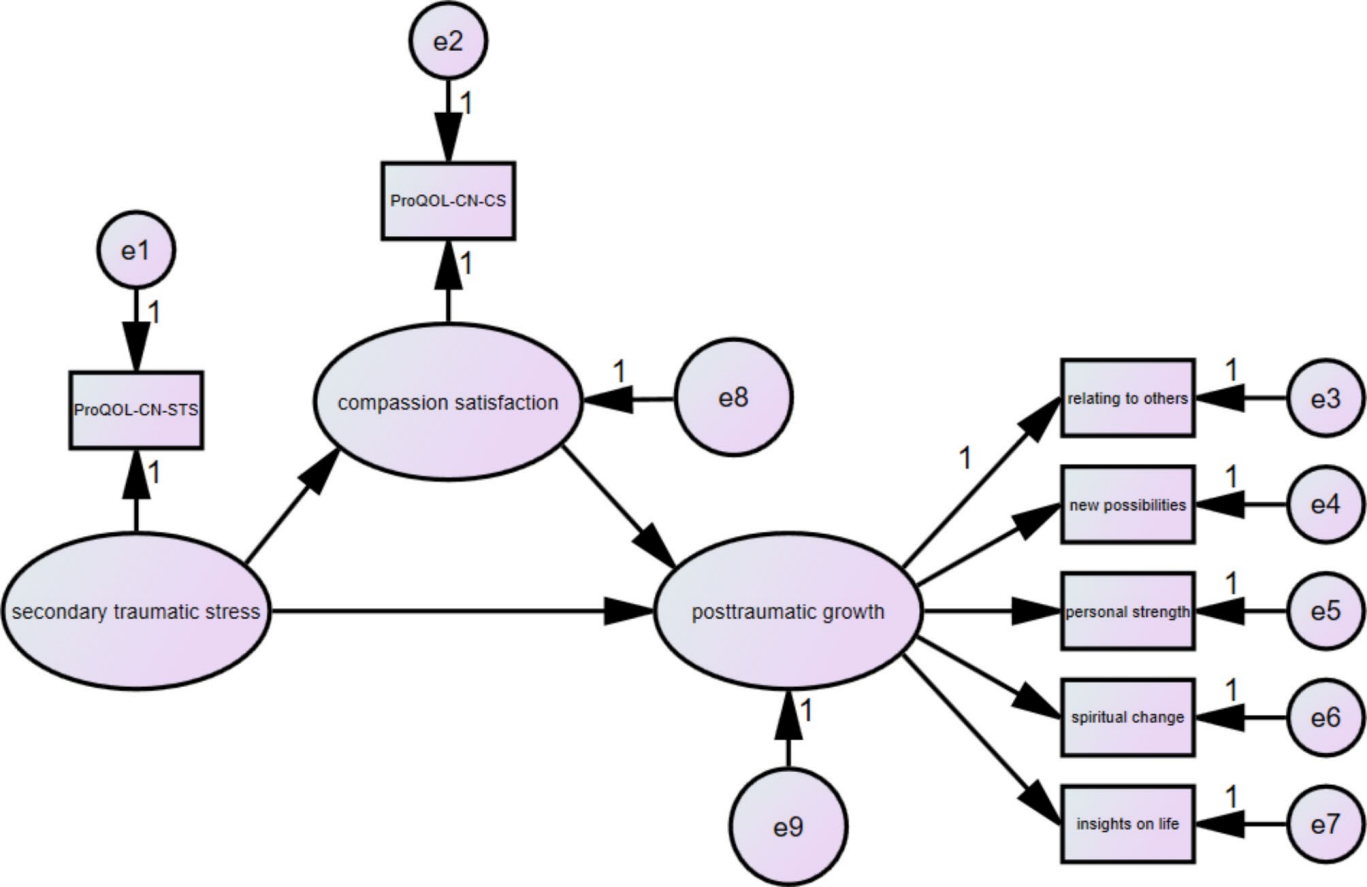
The mediate effect model of compassion satisfaction between secondary traumatic stress and posttraumatic growth

The hypothetical model was tested by structural equation modeling, which includes three latent constructs (secondary traumatic stress, compassion satisfaction and posttraumatic growth) and seven observed variables (Fig. 1). In this model, the fitting degree of the model was appropriate as follows: *χ*^*2*^/df = 3.006, TLI = 0.978, CFI = 0.986, IFI = 0.986, RFI = 0.967, NFI = 0.980, RMSEA = 0.078. Furthermore, all factor loads of indicators on latent constructs were significant (*p* < 0.05), indicating that all latent constructs were well represented by their indicators.

The mediating roles of compassion satisfaction were examined by 5000 bootstrap analyses with 95% confidence intervals. As shown in Table 3, secondary traumatic stress had a significant direct effect on compassion satisfaction (*β* = 0.160, *p* = 0.007) and posttraumatic growth (*β* = 0.100, *p* = 0.030). The direct effect of compassion satisfaction on posttraumatic growth was 0.558 (*p* = 0.001). The indirect effect of secondary traumatic stress → compassion satisfaction → posttraumatic growth was 0.089 (*p* = 0.007), which suggested that compassion satisfaction partially mediates between secondary traumatic stress and posttraumatic growth.

**Table 3:** Direct and indirect effects for the model (N = 330)

Model pathways	Standardized effect (*β*)	*95% CI*	*p*
Direct effect			
Secondary traumatic stress → compassion satisfaction	0.160	(0.054, 0.260)	0.007
Compassion satisfaction → posttraumatic growth	0.558	(0.478, 0.630)	0.001
Secondary traumatic stress → posttraumatic growth	0.100	(0.010, 0.193)	0.030
Indirect effect			
Secondary traumatic stress → compassion satisfaction → posttraumatic growth	0.089	(0.028, 0.149)	0.007

## Discussion

In this study, we found that the scores of secondary traumatic stress and compassion satisfaction of newly graduated nurses were 27.11 ± 4.94 and 31.89 ± 6.22, respectively, which were at a moderate level, while their posttraumatic growth score was 56.47 ± 20.41, which was in the low level. Compared with previous studies, the level of secondary traumatic stress of newly graduated nurses is obviously higher, but the level of compassion satisfaction and posttraumatic growth are lower [8, 26]. The reason for this result may be that newly graduated nurses have just come into contact with clinical nursing and lack of work experience, so they can not easily view and cope with work stress, while the slow role transformation also makes nurses unable to clearly find their own position in clinical nursing work and feel the pleasure and achievement brought by helping others [27]. In addition, some studies pointed out that nurses with older age and longer working years had more nursing experience, could better deal with emergencies of patients and reflect afterwards, and their posttraumatic growth level was higher [28, 29]. In contrast, newly graduated nurses lacked both knowledge reserve and practical ability, and their posttraumatic growth level was relatively low [30].

This study revealed that newly graduated nurses who worked hours per day > 8 h had higher secondary traumatic stress level, while newly graduated nurses who slept hours per day > 8 h had lower secondary traumatic stress level, higher compassion satisfaction level and posttraumatic growth level. On the one hand, long working hours and multiple consecutive shifts may increase the risk of nurses’ burnout and secondary traumatic stress, cause occupational injury, and then affect the overall quality of nursing [31, 32]. On the other hand, nurses with sufficient sleep time are more likely to maintain good physical and mental state, better recover energy, face various emergencies at work, so as to reduce secondary traumatic stress level, improve compassion satisfaction level, and ultimately promote posttraumatic growth [8, 10]. In addition, the results of this study suggested that newly graduated nurses who had workplace violence experience had a higher level of secondary traumatic stress and a lower level of posttraumatic growth, which is consistent with the findings of Zeng et al. [16]. Even though nurses had invested a lot of energy in nursing work, they might still suffer from workplace violence, which will cause compassion fatigue of nurses and aggravate their secondary traumatic stress, and their posttraumatic growth level might also be affected [33].

Furthermore, this study also verified the positive correlation between secondary traumatic stress, compassion satisfaction and posttraumatic growth of newly graduated nurses, and clarified more specific information among the three through structural equation model. It can be seen from the results that the three path coefficients including secondary traumatic stress → posttraumatic growth, compassion satisfaction → posttraumatic growth and secondary traumatic stress → compassion satisfaction → posttraumatic growth are statistically significant.

### Hypothesis 1

proposed that secondary traumatic stress has a direct effect on posttraumatic growth, which was confirmed by this study and is consistent with prior reports [15, 34]. Chen et al. believed that when nurses’ traumatic stress experience was maintained over time, they might take some coping measures to make themselves reconstruct this experience cognitively, thus producing positive results, such as posttraumatic growth [35]. Another study showed that when nurses faced high-intensity secondary traumatic stress, their inner painful experience was triggered, while in order to obtain self-change and detachment, they could stimulate self-reflection on the existing situation, and posttraumatic growth followed [8].

### Hypothesis 2

that compassion satisfaction has a positive effect on posttraumatic growth was also confirmed by this study. Zeng et al. proposed that compassion satisfaction was one of the important factors affecting the posttraumatic growth of nurses, and the higher the level of compassion satisfaction of nurses, the more they could obtain energy from nursing work, so as to handle various events more calmly and deal with psychological stress, thus promoting the improvement of their posttraumatic growth level [16].

Besides, the research results on the relationship between secondary traumatic stress, compassion satisfaction and posttraumatic growth allowed us to corroborate hypothesis 3, that is, secondary traumatic stress can not only directly affect posttraumatic growth, but also indirectly affect posttraumatic growth through the mediating effect of compassion satisfaction. Previous studies pointed out that although secondary traumatic stress might cause emotional exhaustion of nurses and have a negative impact on nursing work, it might also cause nurses to rethink their personal values and promote posttraumatic growth due to the special high-pressure environment [8, 15]. As for the impact of secondary traumatic stress on compassion satisfaction, some studies think it is negative, others think it is positive, and some studies think there is no relationship between the two, which may be related to the background of the respondents and the complex and diverse nursing environment [36–38]. In this study, nurses with higher levels of secondary traumatic stress can obtain a stronger sense of achievement in clinical nursing work and a higher level of compassion satisfaction, which provides the possibility for the occurrence and improvement of posttraumatic growth.

### Limitations

This study has some limitations. First, the cross-sectional design could not test the causal mechanism between secondary traumatic stress, compassion satisfaction and posttraumatic growth. Next, we adopted convenient sampling when collecting data, which may limit the universality of the sample, and whether the research results are applicable to other populations or regions needs to be further verified. Finally, the scales used are self-reported, and the results may be biased.

## Conclusion

As far as we know, this is the first time to explore the prevalence and internal relationship of secondary traumatic stress, compassion satisfaction and posttraumatic growth among newly graduated nurses. The results of this study suggested that the secondary traumatic stress and compassion satisfaction level of newly graduated nurses were at a moderate level, but the posttraumatic growth level was at a low level, which is affected by work hours, sleep hours and workplace violence experience. Based on this, nursing managers should pay attention to the mental health of newly graduated nurses, reasonably arrange shifts and individual working hours, establish or improve the mental health monitoring platform and work-related violence incident reporting system, regularly evaluate the trauma stress experience and various emotional reactions of newly graduated nurses. Moreover, the structural equation model showed that newly graduated nurses’ compassion satisfaction played a mediating role between secondary traumatic stress and posttraumatic growth. Therefore, nursing managers can also help the newly graduated nurses actively adapt to the environment, speed up the role transformation, improve their clinical nursing ability and pressure resistance ability by carrying out relevant induction training, so as to improve their level of compassion satisfaction, and achieve personal growth.

## Data Availability

The data that support the finding of this study are available from the corresponding author upon reasonable request.
