# Correction: Secondary traumatic stress and posttraumatic growth in newly graduated nurses: the mediating role of compassion satisfaction

**DOI:** 10.1186/s12912-023-01681-3

**Published:** 2024-01-09

**Authors:** Li Zeng, Xiangeng Zhang, Guiling Liu, Dong Liu, Lan Li, Man Jin, Xin Li, Jialin Wang

**Affiliations:** 1 Sichuan Nursing Vocational College, Chengdu, Sichuan China; 2 College of Nursing, Chengdu University of Traditional Chinese Medicine, Chengdu, Sichuan China; 3 College of Modern Nursing, Dazhou Vocational and Technicial College, Dazhou, Sichuan China; 4 School of Pharmacy, Chengdu Medical College, Chengdu, Sichuan China; 5 The Third People’s Hospital of Chengdu, Chengdu, Sichuan China; 6 Army Medical Center of PLA, Chongqing, China

**Correction to: BMC Nursing (2023) 22:295**

10.1186/s12912-023-01456-w

Following publication of the original article [1], the authors reported that the affiliations of the author Li Zeng should be one and two instead of one. The author affiliations have been updated.

The original article [1] has been corrected.
